# Synergy Between Low-Cost Chitosan and Polyaluminum Chloride (PAC) Improves the Flocculation Process for River Water Treatment

**DOI:** 10.3390/polym17131822

**Published:** 2025-06-30

**Authors:** Gonzalo De-Paz-Arroyo, Andrea M. Torres-Iribe, Lorenzo A. Picos-Corrales, Angel Licea-Claverie, Grégorio Crini, Evangelina García-Armenta, Diana V. Félix-Alcalá

**Affiliations:** 1 Facultad de Ingeniería Culiacán, Universidad Autónoma de Sinaloa, Ciudad Universitaria, Culiacan 80013, Sinaloa, Mexico; 2 Facultad de Biología, Universidad Autónoma de Sinaloa, Ciudad Universitaria, Culiacan 80013, Sinaloa, Mexico; 3 Facultad de Ciencias Químico Biológicas, Universidad Autónoma de Sinaloa, Ciudad Universitaria, Culiacan 80013, Sinaloa, Mexico; 4 Centro de Graduados e Investigación en Química, Tecnológico Nacional de México/Instituto Tecnológico de Tijuana, Calzada Tecnológico SN, Tijuana 22414, Baja California, Mexico; 5 Chrono-Environnement, Université Marie et Louis Pasteur, 25000 Besançon, France

**Keywords:** chitosan, flocculation, synergistic effects, dual flocculation, river water treatment

## Abstract

Currently, there is a demand for effective flocculant systems that can be used without adverse impact on the environment and health. However, the challenge is to find the minimum dose to achieve the same efficacy as conventional flocculants. One technique involves using a mixture of natural and synthetic flocculants, the synergistic effects of which can enhance treatment efficiency. Thus, this work provides an approach using a low-cost chitosan (CH56)–polyaluminum chloride (PAC) mixture as a flocculant system for river water. Therefore, water quality was monitored in the Tamazula and Humaya rivers, which are sources of water for potabilization plants. According to the results of flocculation tests, the use of the mixture required a lower dosage (0.75 mg L^−1^ of CH56 with 1 mg L^−1^ of PAC; 0.75 mg L^−1^ of CH56 with 2 mg L^−1^ of PAC) than that used with individual flocculants (3 mg L^−1^ of CH56; 5 mg L^−1^ of PAC). Conveniently, the mixture produced larger and more compact flocs, favoring sedimentation kinetics and thus flocculation. Fractal dimension (F_D_) and lacunarity (Λ) from microscopy images were used as indicators of the quality of the flocs formed. In general, CH56 and the mixture performed better than PAC, and the mixture allowed the best removal of the model microplastic (polystyrene). Flocculant mixtures reduced the concentration of copper ions by 58%, of tetracycline by 22%, of microplastics by 80%, and of bacteria by >90%. Hence, the authors believe that this work offers valuable information that could be used for potabilization plants aiming to reduce the dose of PAC and introduce chitosan into their coagulation–flocculation process.

## 1. Introduction

To date, several technologies have been tested to remove suspended solids and particles, including colloids, present in contaminated water and wastewater; these technologies include filtration, centrifugation, coagulation alone or coupled with flocculation, electrocoagulation, flocculation (conventional or electrical flocculation), precipitation coupled with flocculation, flotation, and biological approaches [1]. One of these technologies, coagulation–flocculation, is widely used as a solid–liquid separation process at an industrial scale [2,3]. As an example, the Municipal Board of Drinking Water and Sewage of Culiacan (JAPAC) in Mexico produces 500, 500, 500, 300, and 150 L s^−1^ of drinking water from each of five potabilization plants that utilize a flocculation process using polyaluminum chloride (PAC) [4].

In water treatment, coagulation and flocculation occur in two main successive steps, namely, a destabilization step and an aggregation step. These processes consist of combining insoluble particles and/or dissolved organic matter, organics, inorganics, and microorganisms into large aggregates, thereby facilitating their removal in subsequent sedimentation and filtration stages. The mechanism can be described as follows: (i) charge neutralization, where the coagulant–flocculant neutralizes particles with a surface charge opposite its own; (ii) adsorption bridging, in which each polymer chain adsorbs onto different particles and brings them together; (iii) double-layer compression, where an increase in ionic strength results in the compression of the double layer (reduced repulsive force), leading to destabilization of the colloids; and (iv) sweep flocculation, which involves the enmeshing/sweeping-out of colloidal particles in a growing amorphous precipitate [5,6].

According to a survey by Wei et al., dose, pH, and temperature are factors influencing sludge dewatering. For example, an optimum dose can be selected depending on the dewatering efficiency and economic considerations. Additionally, the surface charges of some flocculant agents and colloidal particles can be affected by pH, which thus affects charge neutralization. Likewise, a high temperature may improve the solubility and activity of polymeric flocculants, resulting in an extended conformation of macromolecules and enhancing bridging flocculation [7]. To date, investigations have demonstrated the efficiency of chitosan as a coagulant–flocculant agent, both in synthetic waters prepared in the laboratory and in raw waters taken from rivers and contaminated effluents [8,9,10,11,12,13]. The main characteristics and properties of chitosan as a flocculant agent are as follows: it is a low-cost product made from a renewable resource (chitin), a non-toxic amino–polysaccharide that is non-corrosive and safe to handle, an eco-friendly and biodegradable substance, and a polyelectrolyte at acidic pH with high cationic charge density; it forms long macromolecular chains favoring bridging of aggregation and precipitation and forms hydrogen bonds; it also acts as an adsorbing and chelating agent, showing outstanding performance (e.g., in terms of removing turbidity, color, metal ions, microalgae, and small organic molecules), producing large flocs, and removing bacteria, viruses and fungi [11,14,15,16]. Compared with conventional chemical flocculants, chitosan also has the advantages of avoiding secondary contamination by metals and efficiency in cold water. Additionally, flocculation using chitosan on an industrial scale could be performed in the same water-treatment plants that currently use commercial PAC applied in liquid formulation [17]. Concerning the synergistic effect of chitosan and inorganic flocculants, the mixture of commercial PAC with chitosan has yielded removal efficiencies of turbidity and dissolved organic matter of around 87% and 63%, respectively [18]. Similarly, the combination of AlCl_3_ and chitosan improves charge neutralization and adsorption bridging abilities compared with the use of the single polysaccharide [19].

The structural characterization of flocs can be carried out by acquiring images followed by their processing using software that provides parameters related to the floc structure. This methodology can be non-invasive and non-destructive for the samples, depending on the implemented protocol. For instance, authors [20] monitored the coagulation–flocculation process, promoted by inorganic and organic coagulants, via an in situ microscope used in conjunction with image analysis. Based on that, the average size and number of flocs were determined as a function of time. Similarly, another research group [21] studied morphological characteristics of activated-sludge flocs by using a digital microscope, examining the relationship between the shape of activated-sludge flocs and their settling/dewatering properties. Thus, image analysis is a standard tool for studying floc structures.

On this topic, several publications have suggested that the use of chitosan derivatives and chitosan-based graft copolymers can increase the effectiveness of flocculation compared to processes using unmodified chitosan [22,23]. However, further modification of this polysaccharide involves additional chemical reactions beyond those implemented to convert chitin to chitosan. On the other hand, most reports describe flocculation experiments using water with a low variety of contaminants (pre-treated water). In this work, a flocculation system involving a low-cost chitosan–PAC mixture was evaluated as a feasible option for the production of clean water from raw river water. Quality parameters regarding the removal of turbidity, color, drugs, metals, microbes, and microplastics were monitored. Also, the interactions of the flocculant mixture with contaminants were explored. The proposed flocculant system is outstanding in terms of efficiency, availability, and dosage, while meeting the requirements of environmental responsibility. This system could help managers overcome the challenges faced by potabilization plants from Culiacan throughout the year, such as high levels of turbidity and color, and the presence of heavy metals and microplastics.

## 2. Materials and Methods

### 2.1. Materials

Low-cost chitosan (CH56, a term assigned by the authors to chitosan of 56 mPa s) was purchased from the company Future Foods (Tlalnepantla, State of Mexico, Mexico), with a degree of deacetylation of 80–91.8%; a viscosity of 56 mPa s; a viscosity-average molecular weight (M_v_) ≈ 51 KDa, as determined from the intrinsic viscosity in CH_3_COOH/water (1% *v*/*v*) [8] using a UBB 25 Cannon-Ubbelohde viscometer; from dynamic light scattering (DLS) using a Zetasizer ZS90 (Malvern Instrument Ltd., Worcestershire, UK), hydrodynamic diameter (D_h_) and polydispersity index (PDI) in CH_3_COOH/water (1% *v*/*v*) were 712 nm and 0.357, respectively; based on laser doppler electrophoresis using the same Zetasizer ZS90, the zeta potential (ζ) was +4.4 mV (at pH 7.4, as for river water samples). Polyaluminum chloride (PAC) was donated by the Municipal Board of Drinking Water and Sewage of Culiacan (JAPAC) and was characterized as a yellow powder containing Al_2_O_3_ at about 30%. Tetracycline (TC, ≥98.0%) was purchased from Sigma-Aldrich (Toluca, State of Mexico, Mexico). Anhydrous cupric sulfate (CuSO_4_, ≥98.0%) was purchased from Jalmek (Nuevo Leon, Mexico). Polystyrene microplastics having a density and diameter of 1.05 g cm^−3^ and 250–425 µm, respectively, were obtained by trituration of commercial drinking cups, followed by sieving. Acetic acid (≥99.7%) was obtained from Sigma-Aldrich Mexico. Distilled water (pH 6.5–8) was obtained from the MARCERLAB Company (Culiacan, Sinaloa, Mexico).

Water Samples were collected in the early morning from the Humaya River (24°49′18.3″ N 107°24′08.5″ W) and the Tamazula River (24°48′45.2″ N 107°23′55.6″ W) in Culiacan, Sinaloa, Mexico. Sample collection was carried out through random sampling. It is relevant to highlight that both urban rivers are used as sources of water by potabilization plants in the city. The monitoring was carried out from April to June in the Humaya River and from September to March in the Tamazula River.

### 2.2. Flocculation Experiments

The conventional jar-test method using a PB700 Standard Jar-Floc Tester (Phipps and Bird, Richmond, VA, USA) with six mixers was used for the flocculation experiments. Both flocculants were dosed in liquid form in aqueous solution starting from a stock solution of 5000 mg L^−1^, and chitosan was prepared by adding 1% *v*/*v* acetic acid to solubilize this flocculant. Trials were carried out with water from the Humaya and Tamazula Rivers. The protocol was as follows: the water was divided into several glass beakers, each containing 500 mL of the previously homogenized water sample. Subsequently, the appropriate dosage of coagulant–flocculant (CH56, PAC, and CH56 + PAC) was added, depending on the planned experiment; using the mixture, PAC was added to all glass beakers, and this step was followed by the addition of CH56 1 min later. Next, the contents of the beakers were stirred vigorously at 100 rpm for 5 min. Then, the mixing speed was adjusted to 60 rpm for 25 min; this step was followed by a settling time of 30 min. The pH of the water was not adjusted prior to the addition of reagents. Samples were then taken from the top of the glass (approximately 100 mL deep) to allow measurement of the different analytical parameters. The effects of the flocculant dose, the chitosan/PAC ratio, and the flocculation time on the removal of turbidity and color were studied. A blank experiment was also systematically performed in the absence of reagent to evaluate the sedimentation of the suspension under similar experiment conditions. The experiments were carried out with replicates.

For experiments to evaluate tetracycline (TC) removal, a concentration of 10 mg L^−1^ of this drug was added to 1 L samples of raw water. The removal was calculated by determining the concentrations before and after the treatment; for that, a calibration curve created with data from different TC concentrations (see Figure S1). For trials involving microplastics, five polystyrene particles were added to each sample (500 mL of river water), and after the tests, a count of the flocculated microplastics was carried out. Studies of copper ion removal were carried out using an initial concentration of 3 mg L^−1^ of Cu^2+^ (from CuSO_4_) in the samples (river water). The concentrations before and after the treatment were recorded from a multiparameter HI83200 (HANNA Instruments, Woonsocket, RI, USA). In both cases, the method for high-range copper was selected. Thereafter, the equipment was calibrated using 10 mL of unreacted sample to set the reading to zero, and then the content of a packet of bicinchoninate HI93702-0 was added to the unreacted sample, with shaking for about 15 s, and finally, the Cu^2+^ content was obtained. Infrared spectroscopy, carried out using a Spectrum 400 (Perkin Elmer Cetus Instruments, Norwalk, CT, USA), was used to investigate the interaction between chitosan and PAC. The powders of CH56, PAC, and the mixture were analyzed; in the case of the mixture, CH56 + PAC were first dissolved and freeze-dried as a single sample.

### 2.3. Water Quality and Floc Exploration

In order to determine the characteristics of the water to be treated, various analyses were conducted on each sample and the following classical parameters were measured: turbidity, color, pH, total dissolved solids (TDS), dissolved oxygen (DO), and photometry. Turbidity was recorded at 25 °C using a 2100P Portable Turbidimeter (HACH, Loveland, CO, USA) with a halogen-filled tungsten-filament lamp. The color of each sample, including in experiments with TC, was obtained from a DR-890 Portable Colorimeter (HACH, Loveland, CO, USA), which was equipped with a beam height of 5 mm and wavelengths of 420, 520, 560, and 610 nm. First, a cuvette filled with deionized water was used to set the reading to zero. Measurements were carried out in triplicate for each sample.

The pH, TDS, and DO of the water samples were measured with an advanced portable multiparameter device (HI9829; HANNA Instruments, Woonsocket, RI, USA) at 25 °C. The concentration of copper ions was estimated at 25 °C as specified above. 

Floc images were acquired with an optical microscope (Primostar 3, Carl Zeiss; Suzhou, China) and with a digital camera Nikon Coolpix B500 (Nikon, China). All experiments and measurements were performed and recorded in triplicate. The methodology employed for floc analysis was as follows: for the analysis of macroscopic flocs, photographs of the bottom of the beakers were taken with a digital camera. To study the microscopic flocs, samples of the wet floc were taken with a micropipette, then placed on a slide and analyzed with a microscope (at 10×). Finally, images were taken with a smartphone Samsung Galaxy (50 Mpx; Samsung Electronics, China) adapted to the light microscope, and the scale bar was taken from the lines of a Neubauer chamber. The measurement of morphometric parameters such as fractal dimension (F_D_) and lacunarity (Λ) allowed the study of floc structures at the macroscopic and microscopic scales; the determination of these parameters was based on the method proposed by García-Armenta et al. [24,25] and was carried out using ImageJ v.1.50d software (National Institutes of Health, USA) and the plugin FracLac 2015Febb5810. In the software, the original images (10×; RGB color) were changed to 8 bit-format images (grayscale) and the brightness and contrast were automatically adjusted. Afterward, the sequence Plugins > Fractal Analysis > FracLac was selected. F_D_ and Λ were calculated using the box-counting method.

### 2.4. In Silico Docking Studies

In silico docking studies were conducted to investigate probable interactions between chitosan and tetracycline (drug designated as a model of emerging contaminant), as detailed in the supplementary material file. Briefly, the AutoDock Vina 1.1.2 program was used to determine the binding modes between the compounds. For the validation of the docking interactions and further analysis, the PDBQT output file from the docking was processed using the bioinformatics platform PYMOL 2.0. The 3D-structure model of chitosan (target molecule) was generated using the ChemDraw 16.0. The ligand for this study was tetracycline, and its 3D structure was obtained using ChemBio3D Ultra 16.0.1.4 software. The binding mode with the best energy affinity was selected; the more negative this value, the greater the binding affinity.

### 2.5. Bacterial Culture

Trials were performed after flocculation using samples from the Humaya River and different flocculant systems at optimal dosage. The samples, which included both raw water and treated water (1/100, 1 mL of sample, and 99 mL of sterile water), were filtered under vacuum using cellulose acetate membranes (0.45 µm, PALL Corporation, Michigan, USA) and pre-sterilized glass materials. Subsequently, the membranes were placed on M-Endo-LES agar as culture media (from the MARCERLAB Company; Culiacan, Sinaloa, Mexico) for the identification of *E. coli* and total coliforms. The incubation was completed at 37 °C for 24 h. For colony counting, red–pink colonies were considered to represent total coliforms and bright metallic green were counted as *E. coli*. The results were expressed as colony-forming units per 1 mL of the sample (CFU mL^−1^). The experiments were carried out in triplicate.

### 2.6. Statistical Analyses

The one-way ANOVA test was applied for the comparison of means, and Tukey’s test was applied for comparison of multiple means with a control, where it was required. Both tests were applied with a significance level of *p* < 0.05 using the IBM SPSS Statistics (IBM.SPSS.23) software.

## 3. Results and Discussion

### 3.1. Monitoring of River Water Quality 

**Figure 1:**
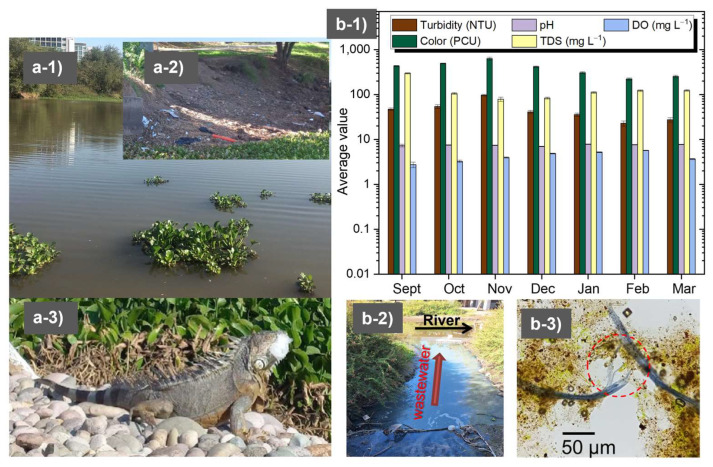
Images showing the appearance of the water (**a-1**), pollution derived from the construction industry (**a-2**), and the impact of plastic pollution on iguanids (**a-3**) in the Humaya River. Images showing the results of monthly monitoring of water quality (**b-1**), wastewater containing detergents discharged into the river (**b-2**), and some of the apparent plastic found in a water sample (**b-3**) from the Tamazula River. Source of photographs: corresponding author.

**Figure 2:**
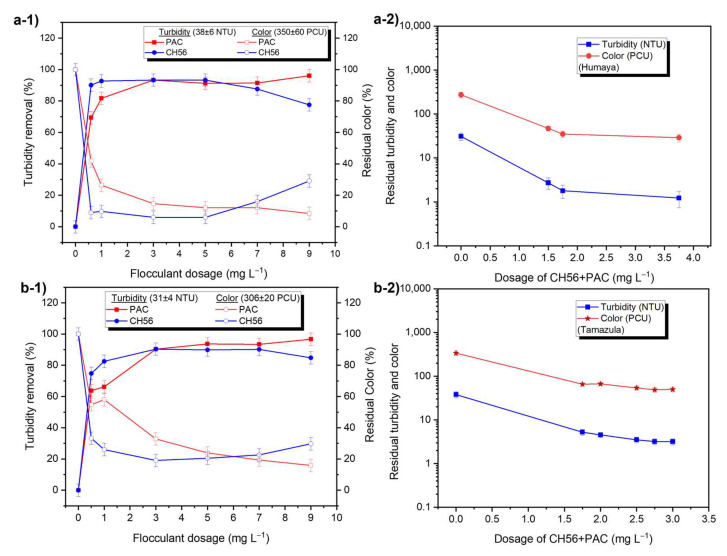
Effect of flocculant dosage on the removal of turbidity (filled symbols) and color (empty symbols) in the Humaya River (**a-1**,**a-2**) and the Tamazula River (**b-1**,**b-2**).

**Figure 3:**
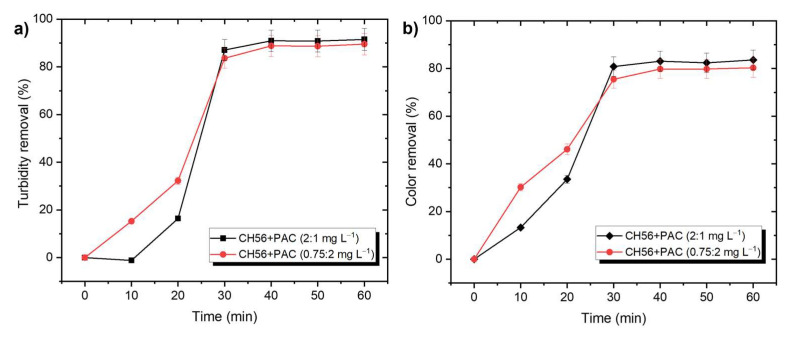
Effectiveness of chitosan + PAC mixtures for (**a**) turbidity and (**b**) color removal at different time, shown using samples from Tamazula River.

Pollution triggers chemical and biological changes in rivers, underscoring the need for regular monitoring, particularly when these water bodies are sources for the production of clean water [26]. In this work, both rivers studied (Humaya River and Tamazula River) receive contaminants from the urban environment and provide water for potabilization plants. Hence, their monitoring is essential to evaluate water quality and estimate the required flocculant dosage. Figure 1 shows evidence of the appearance of water (a-1), pollution derived from the construction industry (a-2), and the impact of plastic pollution on *Iguanidae* lizards (a-3) in the Humaya River. In recent years, the urban river called “Tamazula” has experienced the negative effects of the growth of the city, with the construction of new commercial and housing complexes near the riverbed. Figure 1(a-1) is a photograph taken on a day of high turbidity when there was also a large proliferation of water lilies. As can be seen in Figure 1(a-2), a large amount of residual plastic was detected on the riverbed, and this plastic was inevitably incorporated into the water flow during the rainy season. However, while the plastic material was present, evidence of damage to the fauna was observed, specifically to the *Iguanidae* lizards that represent one of the main attractions for visitors to the river. During that day of monitoring, it was found that an iguanid completely swallowed a fraction of plastic material (Figure 1(a-3)). Additionally, images of representative microorganisms and microalgae present in the Humaya River were obtained, such as *Anabaena* spp. which is a benthic cyanobacteria in rivers and produce cyanotoxins affecting the aquatic food webs [27] Rotifera of the Family *Lecanidae* [28], and images of protists within the taxon *Heliozoa* [29]. Among microalgae, the following were found: species belonging to the genus *Leptocylindrus* [30,31], members of the genus *Pinnularia* [32], species of diatom belonging to the genus *Cyclotella* [33], *Rhopalodia gibba* [34,35], and algae belonging to the genera *Trachelomonas* sp. and *Strombomonas* sp. [36] (Figure S2). Also, monitoring was carried out between the months of April and July (before and at the beginning of the rainy season, Figure S3); the first relevant rain was recorded in the first half of June and was marked by increasing turbidity (from ~30 NTU to ~100 NTU) followed by partial decontamination of the river, which resulted in lower values of turbidity and color in July. Low levels of dissolved oxygen corresponded to a dramatic increase in water lilies and microalgae during the rainy season, and that could have serious implications for aquatic life. TDS (from 138 to 167 mg L^−1^) and pH (around 7.4) did not show significant variation during the sampling period. In the Tamazula River (Figure 1(b-1,b-2,b-3)), turbidity and color values generally decreased as the main rainy period ended. The highest turbidity values were close to 100 NTU, and the lowest values were between 25 and 30 NTU (Figure 1(b-1)). pH was the only parameter that did not show significant variation (remaining around 7.5). Figure 1(b-2) shows evidence of the discharge of untreated wastewater directly into the river; this wastewater originated from the storm drain of Culiacan and contained detergents. Furthermore, Figure 1(b-3) shows material with the appearance of microplastics that was found in the sediment and is similar to the microplastics extracted by other authors from other water samples [37]. The parameters that differed between the rivers were turbidity, color, DO, and TDS. Currently, the high levels of color and turbidity during some days of the year represent a challenge for the Culiacan Drinking Water Board, as they result in greater consumption of PAC; this leads to the need for a more efficient flocculation system.

### 3.2. Removal of Turbidity and Color from River Water Samples

Figure 2 shows the effect of flocculant dosage on the removal of turbidity and color from raw water (Humaya and Tamazula rivers). Turbidity removal for Humaya River samples was slightly higher than 90% at CH56 concentrations from 0.75 to 5 mg L^−1^, and a lower removal was reached with dosages of 7 and 9 mg L^−1^ (Figure 2(a-1)). On the other hand, the turbidity removal increased as the dosage of PAC increased, especially at dosages between 3 and 9 mg L^−1^, which yielded > 90%. Similarly, the residual color was plotted after the flocculation tests, with results showing that CH56 at 3 and 5 mg L^−1^ led to less than 10% of the original, while PAC reduced the color to < 20% of the original values when concentrations from 3 to 9 mg L^−1^ were used. As is well known, inorganic PAC is currently used in water treatment plants; therefore, a mixture involving the chitosan–PAC system could be suitable in terms of effectiveness, availability, and cost of materials. Figure 2(a-2) shows turbidity and color values after the flocculation trials, in which the following combinations of CH56 + PAC concentrations were used: 0.75 mg L^−1^ + 0.75 mg L^−1^, 0.75 mg L^−1^ + 1 mg L^−1^, and 0.75 mg L^−1^ + 3 mg L^−1^. Compared to similar dosages of PAC alone or of chitosan alone, the mixture improved turbidity and color removal at, for example, a total concentration of 1.75 mg L^−1^ (turbidity ~1.8 NTU; color of 35 PCU). The effectiveness is enhanced by increasing the dosage of the mixture, which was similar to that seen with PAC. From similar experiments, authors [9] have also reported that compared with PAC alone, the addition of a low amount of chitosan can improve flocculation performance. A comparable trend was seen with samples from the Tamazula River (Figure 2(b-1)); in this case, the best turbidity removal using CH56 was attained between 3 and 7 mg L^−1^, with these concentrations resulting in removal slightly higher than 90%, while a decrease in turbidity removal was obtained at 9 mg L^−1^ of CH56. In the case of PAC, turbidity and color removal were again improved at higher dosages of flocculant. When the flocculant dose was increased from 1 to 5 mg L^−1^, an increase in the number of flocs formed by PAC was observed. With CH56, no difference in appearance was observed between the flocs formed at a dose of 3 mg L^−1^ and those formed at a dose of 5 mg L^−1^, while the flocs formed at 1 mg L^−1^ were slightly different (see Figure S4). For the mixture (Figure 2(b-2)), the following dosages of CH56 + PAC were used: 0.75 mg L^−1^ + 1 mg L^−1^, 1 mg L^−1^ + 1 mg L^−1^, 2 mg L^−1^ + 0.5 mg L^−1^, 0.75 mg L^−1^ + 2 mg L^−1^, and 2 mg L^−1^ + 1 mg L^−1^. At a total concentration of 2.75 mg L^−1^, turbidity of around 3.2 NTU and color of 49 PCU were recorded. Based on several experiments, it was determined that the mixture at 2.75 mg L^−1^ ensures high flocculation efficiency, which changes when 0.75 mg L^−1^ of CH56 or 2 mg L^−1^ of PAC are used individually. Based on the data on residual turbidity and color with flocculation at 5 mg L^−1^ of PAC (1.8 NTU, 65 PCU), 3 mg L^−1^ of CH56 (3.4 NTU, 64 PCU), and 2.75 mg L^−1^ of mixture (3.2 NTU, 49 PCU), the use of the mixture led to residual values closer to the limits (3 NTU, 15 PCU) established by the Mexican Environmental Regulation (NOM-127-SSA1-2021) [38]. Thus, the results confirmed that the mixture of flocculants could be as effective as single flocculants, with the advantages that they overcome the drawback of overdose (floc redispersion can occur when chitosan is used alone) and also avoid the excess of aluminum associated with high concentrations of PAC. According to a report in the literature [18], polymeric Al species in PAC can weaken the repulsive force between colloids (natural organic matter) by neutralizing their negative surface charge. After the surface charge is neutralized, the chitosan (surface charge close to zero at the pH of water samples, ζ = +4.4 mV) can further speed up the interparticle bridging together with the colloidal Al species (colloidal hydroxides) from PAC, triggering the attachment of more colloids. In addition, the amino and hydroxyl groups in chitosan can promote binding to free Al species, effectively reducing their presence in the treated water.

This study also explored the effectiveness of chitosan + PAC mixtures at different times of the flocculation process, using water samples from the Tamazula River (Figure 3). In this case, the data showed that when it was added during the first 20 min, the mixture (0.75 mg L^−1^ + 2 mg L^−1^) resulted in better removal compared to the mixture with a higher content of chitosan (2 mg L^−1^ + 1 mg L^−1^); however, the final efficiency was similar with both systems. Therefore, considering the amount that would be used on an industrial scale, the mixture with lower chitosan content was selected for future experiments. As a reference, Figures S5 and S6 illustrate the turbidity and color removal profiles as a function of time using PAC (5 mg L^−1^) or CH56 (3 and 5 mg L^−1^). No significant difference was observed between the results. Compared to PAC and CH56, the mixture (0.75 mg L^−1^ + 2 mg L^−1^) presented better initial performance, but similar results were obtained from all these systems after 40 min.

### 3.3. Study of Floc Formation Using Optimal Dosage

**Figure 4:**
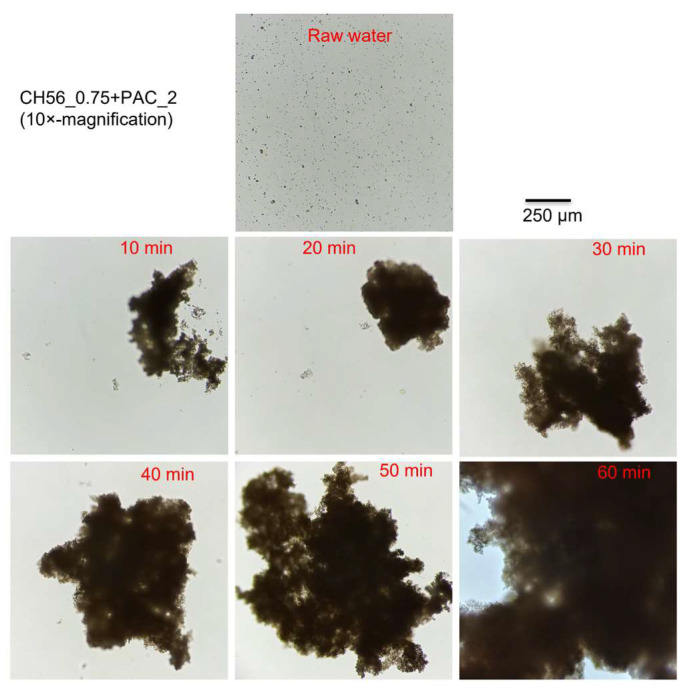
Floc formation at different times using a CH56 + PAC mixture and samples from the Tamazula River (70 NTU).

An additional experiment focused on monitoring floc growth was carried out for each flocculant system. Since some authors have found that the effect of particle concentration on floc sizes is not very pronounced in jar tests [39], with the aim of detecting the effect more clearly, the tests were carried out when the water samples from the Tamazula River presented turbidity values close to 70 NTU. The concentrations of flocculants were adjusted to the turbidity value using the following dosages: mixture chitosan + PAC (0.75 mg L^−1^ + 2 mg L^−1^) (Figure 4), PAC (5 mg L^−1^) (Figure S7), and CH56 (5 mg L^−1^) (Figure S8). For the concentrations used, the flocs formed with the mixture were considerably larger than those obtained with PAC or CH56, while the flocs formed with CH56 were slightly larger than those that formed with PAC at different times. In general, for all systems, continuous growth was observed as time increased. Furthermore, this approach using the mixture might reduce sedimentation time on an industrial scale and favor better management of waste sludge. The behavior of the flocculant mixture was further investigated using the software ImageJ v.1.50d, which was utilized to evaluate the area of the flocs formed at different times (from 0 to 50 min, see Figure S9). After 10 min, a significant increase in area compared to the areas of flocs in raw water was observed, and after 20 min, a constant increase was detected. Therefore, an additional data treatment was carried out in the form of data fitting, finding a linear growth (R^2^ = 0.98) in area during floc formation between 20 and 50 min (see Figure S10). The sample taken at 60 min was not considered because it was taken after sediment consolidation. According to Figure S9 and as described in the literature, floc size first increased and then reached a steady state (from 10 to 20 min), which may be governed by the balance between aggregation and breakage of flocs; specifically, the floc size can remain constant when the breakage and aggregation of flocs are balanced under mechanical stirring [40,41]. Eventually (after 20 min), aggregation may effectively level off due to the adhesion produced by the flocculant mixture, as later demonstrated in experiments with microplastics. Nevertheless, mechanical stirring was stopped after 30 min.

Regarding the evolution of flocculation with time using the mixture, it may be supposed that floc growth evolves step-wise, considering that PAC was the first flocculant added: (i) small aggregates are formed from particles of organic matter after electroneutralization; (ii) they grow to form large flocs, a process helped by the bridging interaction of chitosan together with the colloidal Al species [18,42]. From these experiments, it was found that the synergy between chitosan and PAC promotes accelerated floc growth in a short time.

**Figure 5:**
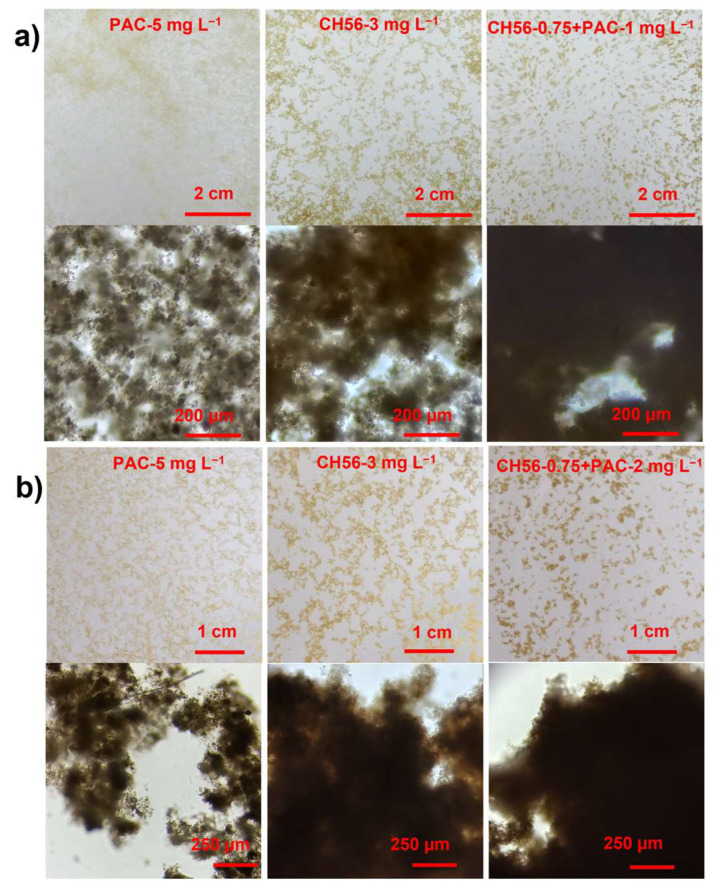
Macroscopic and microscopic images (10×-magnification) of flocs formed with the optimal dosage of each flocculant system, produced using samples from the Humaya River (**a**) and the Tamazula River (**b**).

Figure 5a shows the macroscopic images captured from the treatment with samples from the Humaya River; with PAC at 5 mg L^−1^, the flocs obtained were well dispersed and smaller, while with CH56 at 3 mg L^−1^, the flocs were slightly more compact and larger in size; conveniently, with the CH56 + PAC mixture (0.75 mg L^−1^ + 1 mg L^−1^), the flocs were more compact and larger compared to those produced with the other flocculants. Evidence from the microscopic images showed clearly that the mixture contributed to the formation of more compact flocs. In the case of samples from the Tamazula River (Figure 5b), in the macroscopic and microscopic images, similar trends were observed, where the most dispersed and least compact flocs were obtained using PAC (5 mg L^−1^) and the most compact flocs were formed with the CH56 + PAC mixture (0.75 mg L^−1^ + 2 mg L^−1^). Thus, the images of samples from both rivers showed the same trend, evidencing the advantages of using the mixture of flocculants for floc formation. To obtain representative data, the images were further processed via digital image analysis, as discussed below.

### 3.4. Flocculation Trials with Samples of Different Turbidity and Color Values Using CH56 + PAC

Table 1 presents a comparative study of turbidity and color removal using different samples from the Tamazula and Humaya Rivers and chitosan + PAC mixtures as a flocculant system. In the case of the mixture of 2.75 mg L^−1^ used for Tamazula River samples; it was observed that at an initial turbidity of 18 NTU, turbidity removal reached 80.6%; this value was slightly lower than that obtained with samples with higher turbidity; since for studies carried out from the sample with 29 NTU and 292 PCU to the sample having 77 NTU and 736 PCU as starting values, turbidity removals > 90% were obtained. In the case of color removal, no significant difference was registered, as the results for the flocculant mixture were between 83.2% and 87.8%. However, in analysis of the turbidity and residual color values, the turbidity values fell in a narrow range between 2 and 6 NTU, but the residual color values increased linearly (R^2^ = 0.99 from initial color vs. residual color) with increasing values of initial color. For trials conducted with the mixture of 1.75 mg L^−1^ and samples from Humaya River, residual turbidity and turbidity removal fell within the ranges of 1.2–6.9 NTU and 86.7–92.1%, respectively. Turbidity removal for this river was similar regardless of initial turbidity. For color removal, no significant difference was observed, with all trials yielding values that fell in a narrow range from 84.6% to 88%. Nevertheless, the residual color increased linearly (R^2^ = 0.96) as the initial color increased; thus, this trend was confirmed with samples from both rivers. It is relevant to highlight that the mixture yielded residual values close to/lower than the limits of turbidity (3 NTU, 15 PCU) established by the Mexican Environmental Regulation (NOM-127-SSA1-2021). For the Humaya River samples with initial values of 9 NTU and 81 PCU, both the final turbidity and color met the requirements established in NOM-127-SSA1-2021 without the need for additional treatments for water clarification. The monitoring data indicated that the turbidity values for river water in the absence of rain are less than 45 NTU. Therefore, this mixture, which is highly effective, may be appropriate for use by the Municipal Board of Drinking Water and Sewage of Culiacan (JAPAC) in Mexico. In this case, no significant infrastructure changes would be required to support the flocculation process, since the mixture can be dosed in its liquid form as is currently done with the PAC.

### 3.5. Fractal Dimension (F_D_) and Lacunarity as Indicators of the Quality of the Floc

**Figure 6:**
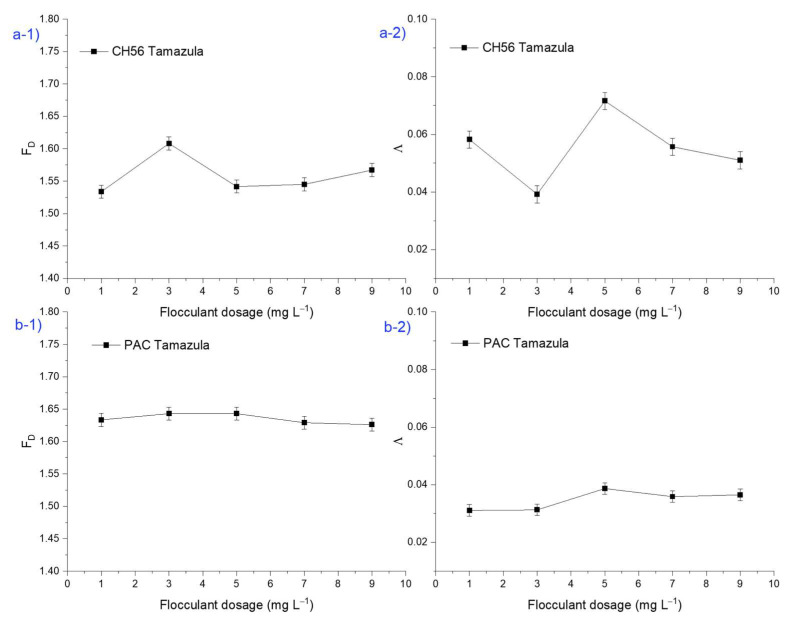
Plots of fractal dimension (F_D_) and lacunarity (Λ) of macroscopic floc images from flocculation performed using (**a**) chitosan and (**b**) PAC individually (initial turbidity of 35 NTU).

Figure 6 shows the results of fractal dimension (F_D_) and lacunarity (Λ) from macroscopic floc images, with results obtained from trials carried out with water from the Tamazula River and using chitosan and PAC individually. For the study involving F_D_ (Figure 6(a-1)) at different dosages of CH56, it is important to highlight that at a dosage of 3 mg L^−1^, a fractal dimension value of 1.63 was observed; this value was higher than that obtained at other dosages. This result could indicate that the flocs were more compact, given that according to the literature [43], when fractal aggregates are densely compacted, they are close to Euclidean objects and so have a large fractal dimension, while smaller fractal dimensions result from more branched structures. Also, the authors stated that the more compact the aggregate, the better the sedimentation performance. This result is in agreement with Figure 2(b-1), as the same CH56 dosage led to the best turbidity removal (> 90%). On the other hand, Figure 6(a-2) presents the results for the lacunarity (Λ) of flocs for the same experiment, where the value of lacunarity calculated at 3 mg L^−1^ is lower than those calculated with other dosages of CH56. Hence, in this case, lower Λ values indicated the formation of more uniform and dense flocs. Moreover, low Λ values can indicate higher symmetry [44]. As shown in Figure 6(b-1), when PAC was used, no substantial difference was observed with respect to the values of fractal dimension (F_D_) at different flocculant dosages, which may indicate that the flocs formed had similar morphologies regardless of the dosage used. However, Λ value (Figure 6(b-2)) was higher at dosages of 5 mg L^−1^ than at 1, 7, and 9 mg L^−1^. It is important to note that the Λ data complement the fractal analysis and that data of this type are usually used to discriminate between the same or similar values of F_D_ [25]. In the case of PAC, which operates by a different flocculation mechanism than chitosan, the optimal PAC dosage was found to be 5 mg L^−1^ (Figure 2(b-1)). The bridging mechanism of chitosan leads to the formation of more compact and larger flocs and therefore to a different trend in the values of F_D_ and Λ [45].

**Figure 7:**
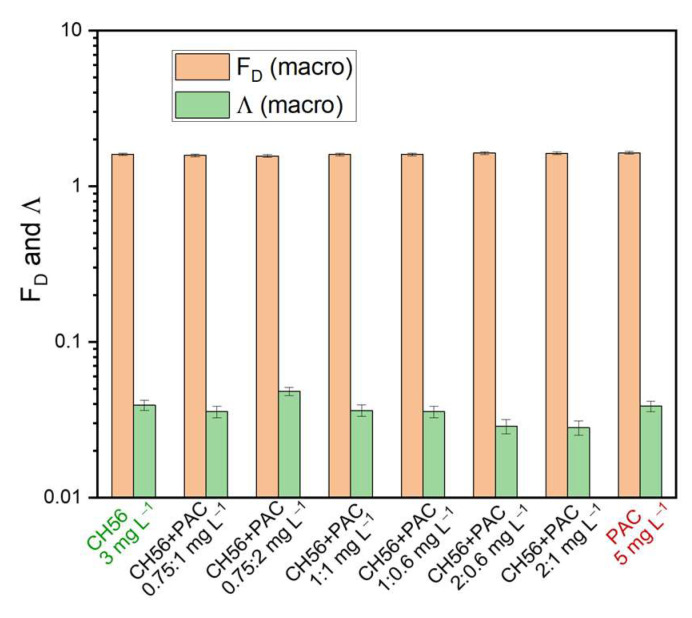
Fractal dimension (F_D_) and lacunarity (Λ) of macroscopic floc images from samples treated using chitosan + PAC mixtures. Water samples were taken from the Tamazula River.

Figure 7 shows the results obtained for F_D_ and Λ from macroscopic images of flocs (considering a complete section of the sediment) using CH56 + PAC mixtures and water samples from the Tamazula River. It is interesting to note that the F_D_ values suggested the production of flocs with similar compactness, regardless of the mass ratios and dosage of the flocculant system. However, except for the mixture formulated at 0.75:2 mg L^−1^, the lacunarity results were slightly lower in samples treated with most of the mixtures compared to samples treated using PAC (5 mg L^−1^) and CH56 (3 mg L^−1^). Furthermore, the samples treated with the mixtures formulated at 2:0.6 mg L^−1^ and 2:1 mg L^−1^ presented the lowest Λ values (about 0.0287 and 0.0281, respectively). As discussed previously, in the case of the flocculant mixture, a combination of mechanisms may act during the flocculation process, resulting in a floc with characteristics different from those obtained using CH56 or PAC individually. Overall, mixtures with a higher CH56 content presented lower Λ values. The image captured during the test with the mixture formulated at 0.75:2 mg L^−1^ gave a higher Λ value on the macroscopic scale (considering a section of the sediment), which can be understood as representing more empty spaces in the image after floc formation.

**Figure 8:**
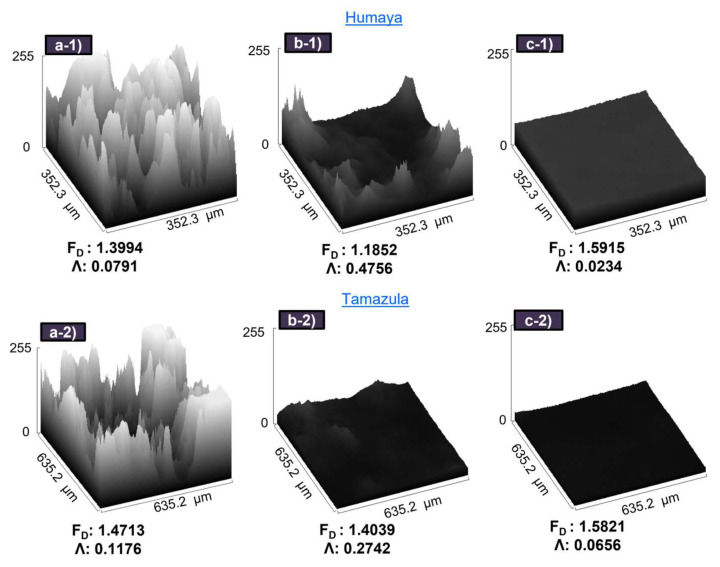
3D surface plot of microscopic images (10×-magnification) of flocs formed with PAC (**a-1**,**a-2**), chitosan (**b-1**,**b-2**), and chitosan + PAC (**c-1**,**c-2**).

Figure 8 presents the values of lacunarity and fractal dimension obtained from microscopic images (10×-magnification) of flocs formed using PAC (5 mg L^−1^), CH56 (3 mg L^−1^), and CH56 + PAC (0.75:1 mg L^−1^, 0.75:2 mg L^−1^). It is relevant to note that the main comparison of the flocs should be conducted between CH56 and the mixture since PAC forms much smaller flocs. For the experiments, flocculation with water from both rivers was explored. When the CH56 + PAC mixture (0.75:1 mg L^−1^) was used to treat water samples from the Humaya River, an F_D_ = 1.5915 was recorded; this value was higher than that those obtained with chitosan and PAC individually; the result was similar (F_D_ value around 1.5821) when the mixture (0.75:2 mg L^−1^) was used to treat water samples from the Tamazula River. It can be highlighted that the trend of data was the same for samples of both rivers, with an inverse relationship between F_D_ and Λ. Specifically, for F_D_, mixture > PAC > CH56, and for Λ, CH56 > PAC > mixture. According to Saxena and Brighu [39], the values of the 2D fractal dimension (microscopic scale) can range from 1 to 2, with lower F_D_ values associated with more porous flocs and higher values suggesting more compact flocs. In an advantage mentioned by Moruzzi et al. [43], more compact aggregates exhibit better performance during sedimentation. This is consistent with the results of flocculation studies carried out at different times, where the mixture (0.75 mg L^−1^ + 2 mg L^−1^) presented better initial performance compared to PAC and CH56, as previously detailed. From the same tests, the Λ results were also assessed, where the mixtures described above (0.75:1 mg L^−1^ and 0.75:2 mg L^−1^) yielded the lowest values of this parameter, at 0.0234 and 0.0656, respectively. Thus, lower Λ values are associated with more uniform and more densely packed flocs [46]. From these trials, it can be suggested that the floc growth using the mixture is improved by the bridging interaction of chitosan together with the colloidal Al species [18]. Specifically, more compact flocs are formed when the mixture is used due to the excellent synergy between PAC and CH56.

### 3.6. Flocculation Tests Adding Contaminants to River Samples

**Figure 9:**
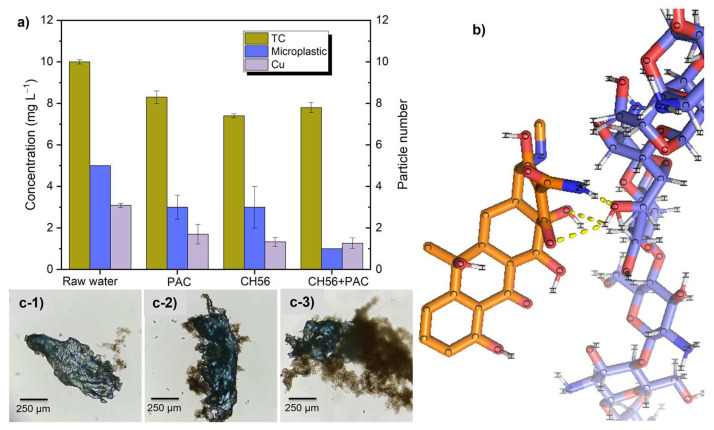
Removal of conventional (Cu^2+^) and emerging contaminants [tetracycline (TC) and microplastics] added to water samples from the Tamazula river: (**a**) concentrations and particle numbers after flocculation experiments; (**b**) probable interactions between chitosan and TC from in silico docking studies; (**c**) images of microplastics with different types of flocs formed with PAC (**c-1**), CH56 (**c-2**), and CH56 + PAC (**c-3**).

Figure 9a presents studies involving the removal of conventional (copper ions) and emerging contaminants (tetracycline and microplastics), which were added to water samples from the Tamazula River. For these tests, dosages of PAC (5 mg L^−1^), CH56 (3 mg L^−1^), and CH56 + PAC (0.75:2 mg L^−1^) were used. Regarding the results obtained when the removal of tetracycline (TC) was evaluated, it can be stated that at an initial concentration of 10 mg L^−1^, all flocculants presented a statistically significant difference in drug removal with respect to the raw water (*p* < 0.001, i.e., *p* < 0.05). In the same way, statistically significant differences were observed when CH56 (*p* < 0.001, i.e., *p* < 0.05) and the mixture (*p* = 0.011, i.e., *p* < 0.05) were compared with PAC. Furthermore, no statistically significant difference (*p* = 0.170, i.e., *p* > 0.05) was found between CH56 and the mixture. Specifically, CH56 led to the best performance, removing 26% of the drug.

The results of the molecular docking study revealed multiple binding modes with different affinity energies (Figure 9b). The most favorable binding mode, with affinity energy of −3.2 kcal mol^−1^, was found between the chitosan structure and TC as neutral molecules (only hydrogen-bonding interactions) and suggested a moderately favorable interaction between these two molecules. This observation supports the potential efficacy of chitosan as a flocculant for TC removal in river water; however, other interactions with less negative affinity energies (−3.0, −3.0, −2.9, −2.9, and −2.8 kcal mol^−1^) were detected. All modes showed a binding capacity between chitosan and TC. During the analysis of interactions between chitosan and TC, a total of three polar bonds were identified, and these were related to hydrogen bond interactions between the -OH groups of the molecules and between the C=O group of the TC and an –OH of chitosan; additionally, a polar bond was observed between the hydrogen of the amino group (–NH_2_) in TC and the oxygen of the –OH group in chitosan. It is known that at a pH around 7.4 (as in river water samples), a high percentage of neutral and anionic forms of TC should exist [47]. Therefore, Zhao et al. reported binding energies of −28.3 kcal mol^−1^ due to electrostatic and hydrogen bonding interactions between charged TC and chitosan [48]. These interactions could contribute to the stability of the complex formed by these molecules.

Additionally, the removal of polystyrene (PS) microplastics with an initial concentration of 5 particles in 500 mL of river water was studied. Tukey’s test indicated significant differences in the removal efficiency in a comparison of samples with each of the flocculant systems to the sample with no flocculant (*p* < 0.05). The CH56 + PAC mixture produced the best removal, reducing the particle numbers to 1 PS particle after flocculation; this was demonstrated to be statistically significantly different compared with the results obtained using PAC (*p* = 0.005, i.e., *p* < 0.05) and CH56 (*p* = 0.012, *p* < 0.05). Between PAC and CH56, no statistically significant difference was observed in the removal of PS (*p* = 0.892). This was supported by images of microplastics trapped by flocs produced using different flocculants; from that, the flocs formed by the CH56 + PAC mixture (Figure 9(c-3)) exhibited stronger interactions (better adhesion) with the microplastics compared to the flocs formed with PAC (Figure 9(c-1)) and CH56 (Figure 9(c-2)). Besides, in previous sections, it was discussed that use of the mixture triggered the formation of larger flocs compared to the use of individual flocculants, and Awan et al. suggested that microplastics removal is more favored when the floc size is larger [49]. Hence, the excellent synergy between CH56 and PAC can lead to significantly improved microplastic removal.

As can be seen for the case of copper ion removal, the initial concentration was fixed at 3 mg L^−1^ of Cu^2+^, a value higher than the limit (2 mg L^−1^) established by NOM-127-SSA1-2021. As measured after the flocculation tests, the CH56 + PAC mixture reduced the concentration to 1.27 mg L^−1^ of Cu^2+^ (Cu^2+^ removal of 58%). All flocculants offered statistically significant differences in Cu^2+^ removal compared to the raw water (*p* < 0.005, i.e., *p* < 0.05). However, there was no statistically significant difference between PAC and CH56 (*p* = 0.489, i.e., *p* > 0.05), and CH56 and the mixture (*p* = 0.995, i.e., *p* > 0.05) also performed similarly. As described in the literature [50], the amino groups on the chitosan chain can act as chelating sites to form a chitosan–copper chelate, and when the pH is close to the neutral value, the structure of the chelate can be {[Cu(-NH_2_)_2_]^2+^, 2OH^−^}. Hence, two amino groups from different chains may associate within the same complex. Consequently, part of the copper removal may involve direct precipitation in the form of complexes.

### 3.7. Removal of Bacteria from River Samples

In order to assess the removal of *E. coli* and total coliforms, studies were carried out by comparing raw river water to treated samples with flocculants at optimal dosages [PAC 5 mg L^−1^, CH56 3 mg L^−1^, mixture (0.75 mg L^−1^ of CH56 + 1 mg L^−1^ of PAC)] (Table 2). The results are expressed as colony-forming units per 1 mL of the sample (CFU mL^−1^). The removal of bacteria was evident in the bacterial culture assays. In most experiments, *E. coli* was not detected (*E. coli* removal > 99%) after treatment; the only exception was one experiment with PAC, in which the presence of a colony was recorded. Similar results (*E. coli* rejection of 99%) have also been achieved using chitosan-based thin-film composite nanofiltration membranes [51], which suggests that the process of flocculation assisted by chitosan is equally efficient. Removal of total coliforms was around 93%, 93%, and 84% using PAC, CH56, and the mixture, respectively. From statistical analyses based on Tukey’s test for total coliforms, all treatments showed significant differences (*p* = 0.001, i.e., *p* < 0.05) with respect to untreated water; in comparisons of PAC vs. chitosan, mixture vs. chitosan, and mixture vs. PAC, the *p* values obtained were 0.999, 0.891, and 0.836, respectively. In other words, there was no significant difference in the removal of these microorganisms among the different flocculant systems. Thus, the mixture offers results comparable to those obtained with the flocculants individually and represents an option that is viable as a flocculant system. Previously, Rehn et al. demonstrated that *E. coli* cells can be immobilized using chitosan as a flocculant [52]. As stated by Li and Zhuang, chitosan presents antibacterial activity against *E. coli*, and this activity can be higher for chitosan with lower molecular weight. Also, the higher the deacetylation degree (the more positive the charge) of chitosan, the stronger its antibacterial activity, given that the interaction between this polysaccharide and bacterial cells occurs mainly via electrostatic interactions [53].

### 3.8. Understanding the Synergistic Effect and Chitosan–PAC Interactions

**Figure 10:**
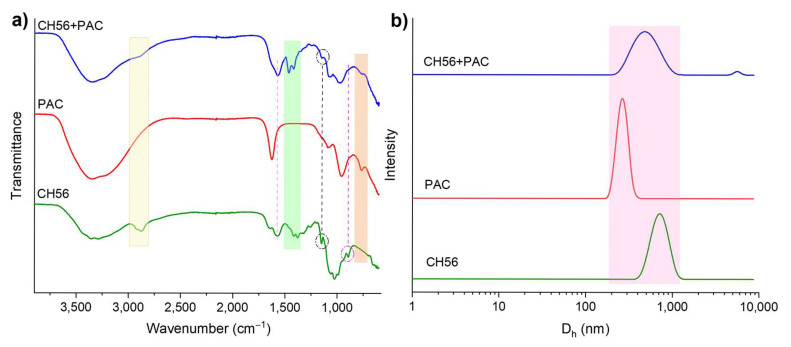
Infrared spectra of CH56, PAC, and mixture (CH56 + PAC, after the freeze-drying process) (**a**). Particle size distributions of CH56, PAC, and CH56 + PAC, obtained by DLS (**b**).

As it was described above, PAC can weaken the repulsive force between colloids by neutralizing their negative surface charge. After that, the chitosan can further speed up the interparticle bridging together with the colloidal Al species from PAC, triggering the attachment of more colloids [18]. In order to investigate the interaction between chitosan and PAC, infrared spectra of powders of CH56, PAC, and CH56 + PAC (the mixture was dissolved, freeze-dried, and analyzed) were recorded (Figure 10a). When the signals from the mixture were compared to those from CH56 or PAC, the transmittance or wavenumber was seen to have changed as a result of the interaction between the polymers. Specifically, the absorption bands at 2920 and 2873 cm^−1^ assigned to the stretching vibration of C-H bonds (sp^3^) in CH56 were barely detected in the mixture. A band at 1575 cm^−1^ related to the N-H vibrations appeared for CH56 and CH56 + PAC. Similarly, the bands between 1455 and 1345 cm^−1^ for CH56 shifted in the mixture (between 1498 and 1370 cm^−1^). The band at 1345/1370 cm^−1^ is usually assigned to C–N stretching. The signal at 1150 cm^−1^ (stretching of the C-O-C bonds) was observed in both samples containing CH56 [54,55]. The band at around 893 cm^−1^ for CH56 was not clearly detected in the mixture; authors have suggested that this signal could arise from a bond involving the N atom [56]. In the comparison of PAC to CH56 + PAC, it was seen that the signal at 1627 cm^−1^ from the PAC spectra was not significant in the mixture spectra, as it was related to the bending vibrations of water absorbed and polymerized in the inorganic polymer. The bands at 1085 and 956 cm^−1^ (vibration of Al–O–Al) were similar in both samples, while the signal at 767 cm^−1^ (attributed to vibrations of Al–OH) was detected only for these two spectra [57,58]. According to the data described above and the literature [59,60], the amino groups of CH56 were attached to the Al atom from PAC, resulting in the changes in the signals obtained for the mixture compared to those obtained for the individual polymers. These analyses confirmed the interaction between the flocculants; this interaction resulted in a synergistic effect that improved the charge neutralization and bridging phenomena. Furthermore, as seen in Figure 10b, the particle size distribution for CH56 + PAC is broad, covering the size range of both flocculants used individually. Hence, the interaction between CH56 and Al species did not produce complex polymer networks larger than CH56, since the low-intensity size distribution detected for the mixture above 4000 nm may be related to the formation of a few polymeric clusters [25].

Regarding current challenges identified in the use of chitosan-based materials for removal of emerging contaminants [61], this research provides findings from variability in ionic composition, organic matter content, and emerging contaminants present in real water matrices. Additionally, the availability of chitin (the primary source of chitosan) is relatively limited [61]; therefore, the mixture of flocculants (CH56 + PAC) represents a suitable option in terms of its effectiveness and availability of materials.

## 4. Conclusions

In general, the monitoring results indicated that the Humaya River showed lower values of turbidity, color and DO and higher values of TDS compared to the Tamazula River. For both rivers, pH values were frequently around 7.5. Furthermore, the ecological damage caused by inadequate practices in the management of plastic waste was evident. Flocculation trials using each flocculant individually for color and turbidity reduction revealed that 3 mg L^−1^ of low-cost chitosan (CH56) and 5 mg L^−1^ of PAC were the optimal concentrations for water samples from the Tamazula River, while 0.75 mg L^−1^ of CH56 and 3 mg L^−1^ of PAC allowed higher performance with samples from the Humaya River. A high effectiveness with mixtures of both flocculants was also reached: the mixtures CH56 + PAC (0.75 mg L^−1^ + 1 mg L^−1^) and CH56 + PAC (0.75 mg L^−1^ + 2 mg L^−1^) were more suitable for water samples from the Humaya River and Tamazula River, respectively. The use of the mixtures yielded residual values closer to the limits (3 NTU, 15 PCU) established by the Mexican Environmental Regulation (NOM-127-SSA1-2021) and better sediment consolidation compared to the use of PAC or CH56 alone. The use of the flocculant mixture overcame the drawback of chitosan, which is that its use usually requires an optimal maximum dosage, with the flocculant being less effective above or below this dose. Additionally, the mixture showed outstanding effectiveness at different initial values of turbidity and color, resulting in removals of around 90 and 86%, respectively, and reduced the concentrations of metal ions (copper ions, by 58%), emerging contaminants like tetracycline (by 22%), microplastics (by 80%), and bacteria (by >90%). Infrared spectroscopy analyses evidenced the interaction between CH56 and Al species, which improved the flocculation process. Thus, the mixture might be appropriate for use on an industrial scale. In this case, no significant changes to the infrastructure used for the flocculation process would be required, since the mixture can be dosed in its liquid form, as is currently done with PAC in water treatment plants.

## Figures and Tables

**Table 1:** Removal of turbidity and color from river samples with different initial turbidity and color using chitosan + PAC mixtures (units: residual turbidity in NTU; turbidity and color removal in %; residual color in PCU).

Tamazula River CH56 + PAC 0.75:2 mg L^−1^	18 NTU; 190 PCU	29 NTU; 292 PCU	40 NTU; 338 PCU	56 NTU; 531 PCU	77 NTU; 736 PCU
Residual turbidity	3.5	2.0	3.2	4.4	6.0
Turbidity removal	80.6	93.1	92	92.1	92.2
Residual Color	32	39	49	65	91
Color removal	83.2	86.7	85.5	87.8	87.6
Humaya River CH56 + PAC 0.75:1 mg L^−1^	9 NTU; 81 PCU	27 NTU; 263 PCU	41 NTU; 405 PCU	53 NTU; 501 PCU	87 NTU; 803 PCU
Residual turbidity	1.2	2.8	4.2	4.6	6.9
Turbidity removal	86.7	89.6	89.9	91.5	92.1
Residual Color	11.0	38.3	63	70.7	96.3
Color removal	86.4	85.4	84.6	86.0	88.0

**Table 2:** Removal of bacteria present in water samples from the Humaya River using PAC, CH56, and CH56 + PAC.

System	*E. coli*	Total Coliforms
	Average Colony Number (CFU mL^−1^)	
Raw water	4 ± 3	42 ± 10
PAC	0	3 ± 0.6
CH56	0	3 ± 2
CH56 + PAC	0	7 ± 4

## Data Availability

Data are contained within the article and Supplementary Materials. The data that support the findings of this study are available on request from the corresponding author.

## Supplementary Materials

The following supporting information can be downloaded at: https://www.mdpi.com/article/10.3390/polym17131822/s1, Figure S1: Calibration curve for tetracycline. River water was treated with each flocculant, and subsequently the different concentrations of the drug were prepared using the treated water as matrix; Figure S2: Images of representative microorganisms and microalgae found in the Humaya River. (a) Cyanobacteria (*Anabaena* spp.), (b,c) Rotifera of the Family “*Lecanidae”*, (d) Protists within the taxon “*Heliozoa*”, (e) Species belonging to genus *“Leptocylindrus”*, (f) Members of the genus *“Pinnularia”*, (g) Species of diatom belonging to the genus “Cyclotella”, (h) *Rhopalodia gibba*, (i) *Trachelomonas* sp., and (j) *Strombomonas sp*; Figure S3: Biweekly monitoring (*in situ* measurements) for 4 months in the Humaya River; Figure S4: Floc formation at different dosages using CH56, PAC, and samples from Tamazula River; Figure S5: Effectiveness of PAC and chitosan for turbidity removal at different time using samples from Tamazula River; Figure S6: Effectiveness of PAC and chitosan for color removal at different time using samples from Tamazula River; Figure S7: Floc formation at different time using PAC and samples from Tamazula River (70 NTU); Figure S8: Floc formation at different time using chitosan and samples from Tamazula River (70 NTU; Figure S9: Area of floc at different time using a chitosan+PAC mixture and samples from Tamazula River (70 NTU); Figure S10: Linear fit of area for floc at different time using a chitosan+PAC mixture and samples from Tamazula River (70 NTU).
