# Supplementary material for: Synergy Between Low-Cost Chitosan and Polyaluminum Chloride (PAC) Improves the Flocculation Process for River Water Treatment
Source: Polymers (Basel). 2025 Jun 30;17(13):1822. doi: 10.3390/polym17131822 (PMC12251655; doi:10.3390/polym17131822)
Supplement: Supplementary file 1 [file polymers-17-01822-s001.zip › polymers-3648435-supplementary.pdf]

Supplementary Material

Synergy between low-cost chitosan and polyaluminium chloride (PAC) improves the flocculation process for river water treatment

Gonzalo De-Paz-Arroyo[1,2], Andrea M. Torres-Iribe[2,3], Lorenzo A. Picos-Corrales[1,2]*, Angel Licea-Claverie[4], Grégorio Crini[5], Evangelina García-Armenta[2,3], Diana V. Félix-Alcalá[3,4]

[1]Facultad de Ingeniería Culiacán, Universidad Autónoma de Sinaloa, Ciudad Universitaria, Culiacan 80013, Sinaloa, Mexico.
[2]Facultad de Biología, Universidad Autónoma de Sinaloa, Ciudad Universitaria, Culiacan 80013, Sinaloa, Mexico.
[3]Facultad de Ciencias Químico Biológicas, Universidad Autónoma de Sinaloa, Ciudad Universitaria, Culiacan 80013, Sinaloa, Mexico.
[4] Centro de Graduados e Investigación en Química, Tecnológico Nacional de México/Instituto Tecnológico de Tijuana, Calzada Tecnológico SN, Tijuana 22414, Baja California, Mexico.
[5]Chrono-environnement, Université Marie et Louis Pasteur, 25000 Besançon, France.

Corresponding author: email: lorenzo.picos.c@uas.edu.mx ; Phone: +52 (667) 7581401

S-2.5 In silico docking

The 3D structure modeling of chitosan (target molecule) was generated using the ChemDraw 16.0. The generated 3D structure was optimized through energy minimization using the MMFF94 (Merck Molecular Force Field 94) method. A molecular force field method used to calculate the energy and geometry of molecules, generating the most stable three-dimensional structure in the UCSF Quimera 1.16 molecule editing and visualization software. Before the docking process, the chitosan structure was prepared by adding polar hydrogen atoms, removing water molecules, adding Gasteiger charge using AutoDock Vina 1.1.2 software to calculate electrostatic interactions between chitosan and tetracycline, and finally, atoms were assigned with AD4 using the AutoDockTools package.

The ligand for this study was Tetracycline, its 3D structure was obtained from ChemBio3D Ultra 16.0.1.4, generating the most stable three-dimensional structure in the UCSF Quimera 1.16 molecule editing and visualization software. To ensure optimal docking, a conformational energy optimization was performed using the MM2 (Molecular Mechanics 2) force field method in the ChemBio3D Ultra16.0.1.4. This involved calculating new positions for each atom to minimize the accumulated potential energy in the 3D structure. Subsequently, ligand preparation involved adding polar hydrogen atoms and Gasteiger charges using AutoDock Vina 1.1.2 software.

A molecular docking study was conducted to explore potential interactions between chitosan and the drug tetracycline. We utilized the AutoDock Vina 1.1.2 program (available at http://vina.scripps.edu/) to determine the binding modes between these molecules. The software

was executed from the command line on the Ubuntu 22.04 LTS Linux operating system, exploring millions of docking configurations. Each output provided several models ranked by binding energy, reflecting the estimated ligand affinity (tetracycline) with chitosan, measured in kcal/mol. The binding mode with the best energy affinity was selected. For this study, a blind docking was performed to investigate possible interactions, meaning that the parameters of the search box and ligand were left at default and random values. Finally, PDBQT files were generated for chitosan and tetracycline, along with the docking result.

For the validation of the docking interactions and further analysis of the previously obtained results, the PDBQT output file from the docking was processed using the bioinformatics platform PYMOL 2.0. PYMOL is a molecular visualization system based on open-source code, maintained and distributed by Schrödinger. PYMOL provided insights into the sizes and locations of binding sites, polar interactions such as hydrogen bonding, hydrophobic interactions, and bond distances as interaction radio >5 Angstrom (Å) from the position of the docked ligand to the target molecule.

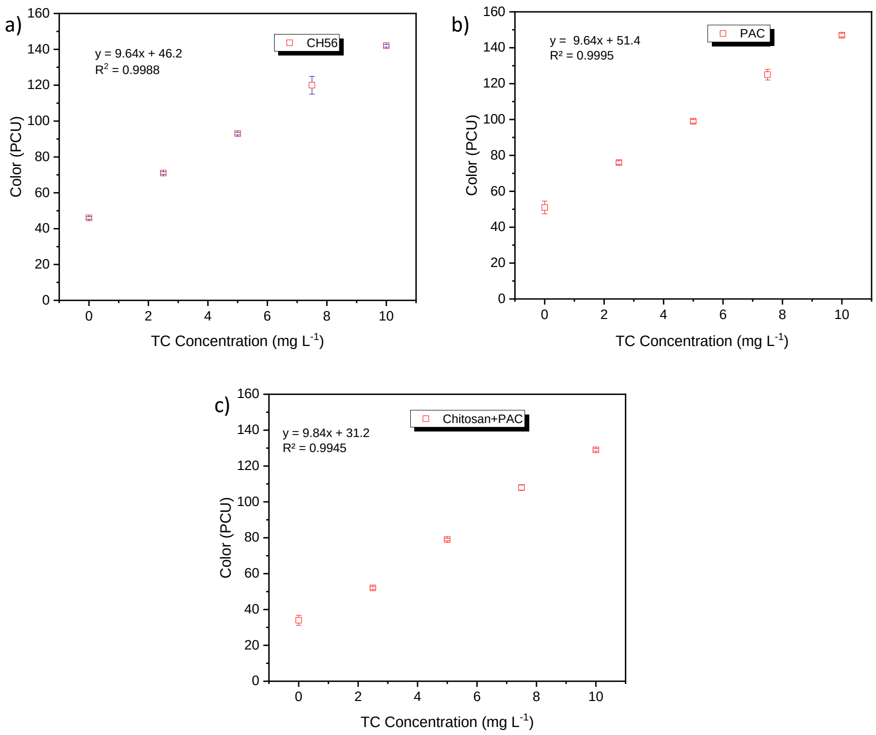

Figure S1. Calibration curve for tetracycline. River water was treated with each flocculant, and subsequently the different concentrations of the drug were prepared using the treated water as matrix.

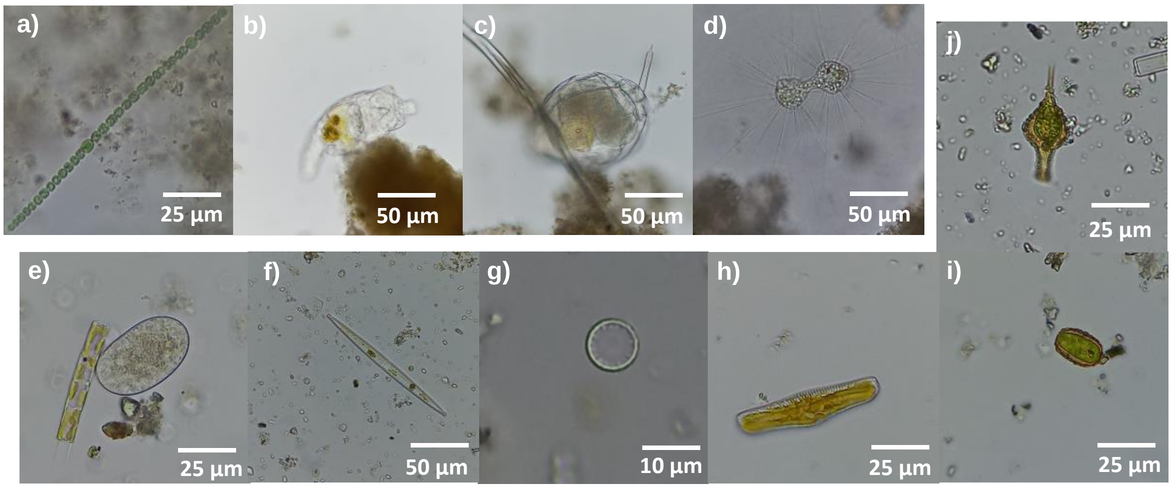

Figure S2. Images of representative microorganisms and microalgae found in the Humaya River. a) Cyanobacteria (*Anabaena spp*.), b-c) Rotifera of the Family "*Lecanidae"*, d) Protists within the taxon "*Heliozoa*", e) Species belonging to genus *"Leptocylindrus"*, f) Members of the genus *"Pinnularia"*, g) Species of diatom belonging to the genus "Cyclotella", h) *Rhopalodia gibba*, i) *Trachelomonas sp.*, and j) *Strombomonas sp.*

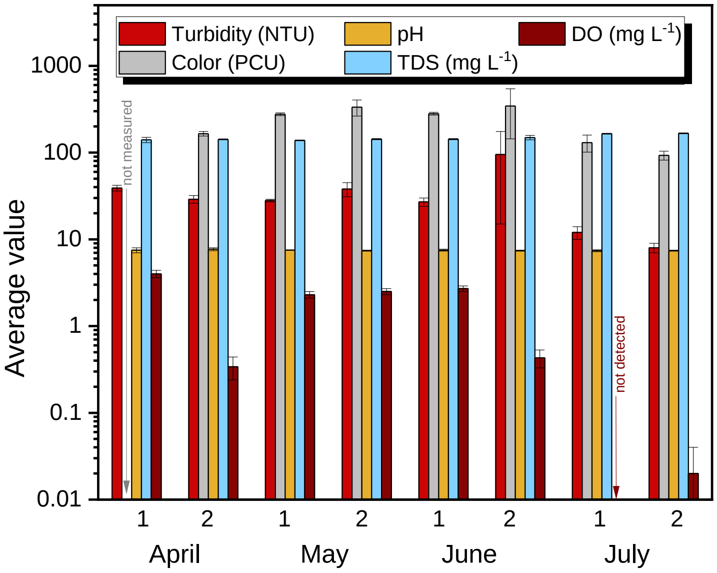

Figure S3. Biweekly monitoring (*in situ* measurements) for 4 months in the Humaya River.

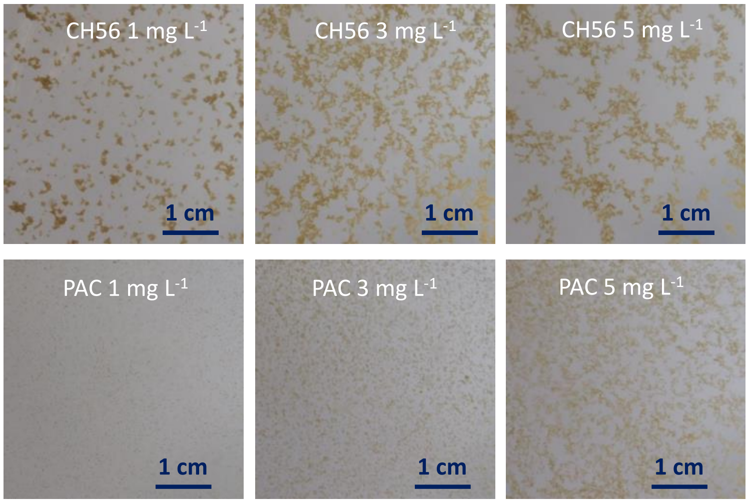

Figure S4. Floc formation at different dosages using CH56, PAC, and samples from Tamazula River.

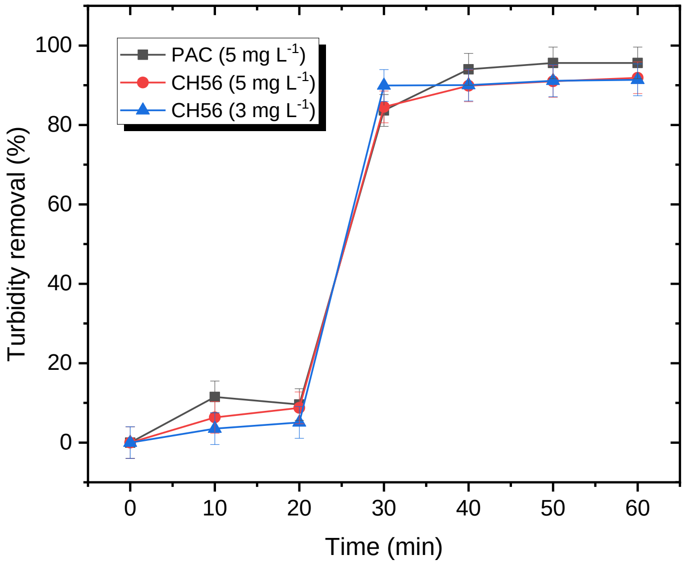

Figure S5. Effectiveness of PAC and chitosan for turbidity removal at different time using samples from Tamazula River.

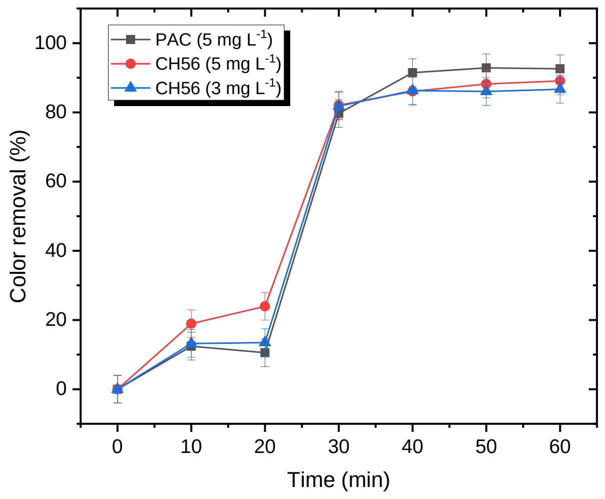

Figure S6. Effectiveness of PAC and chitosan for color removal at different time using samples from Tamazula River.

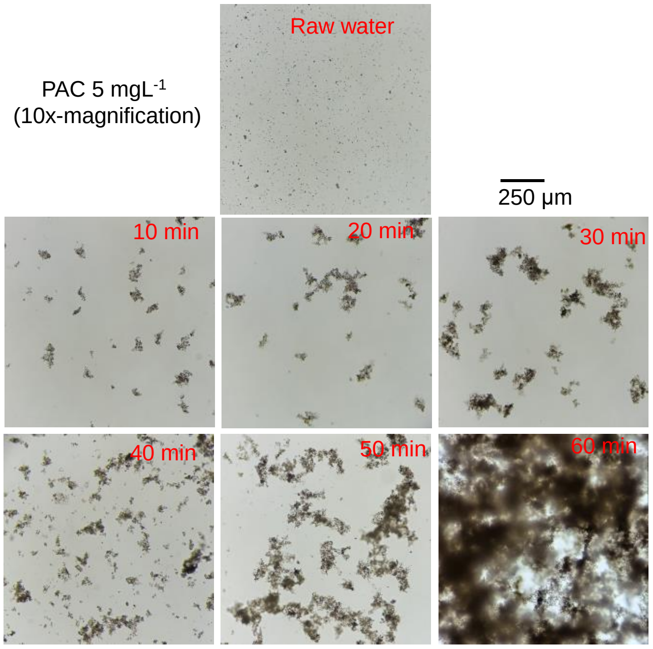

Figure S7. Floc formation at different time using PAC and samples from Tamazula River (70 NTU).

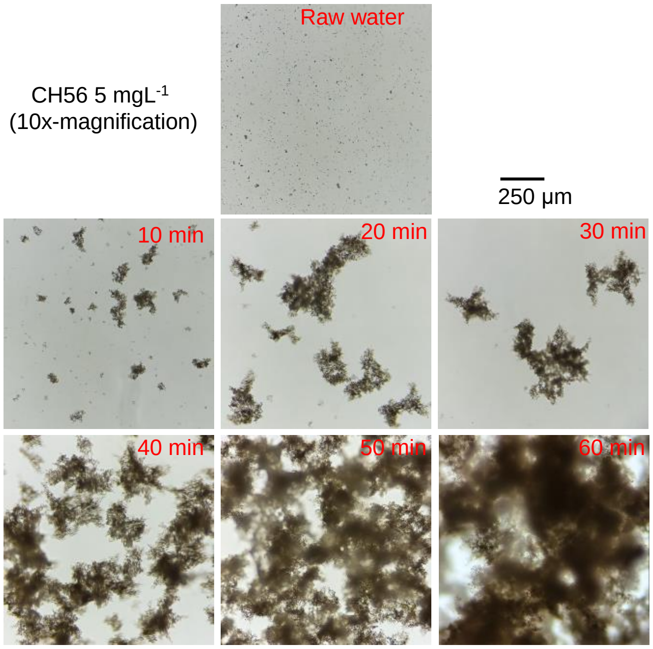

Figure S8. Floc formation at different time using chitosan and samples from Tamazula River (70 NTU).

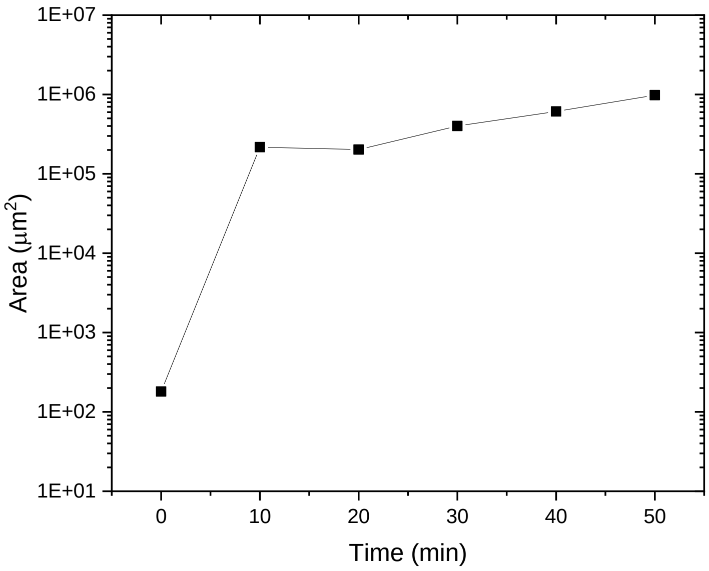

Figure S9. Area of floc at different time using a chitosan+PAC mixture and samples from Tamazula River (70 NTU).

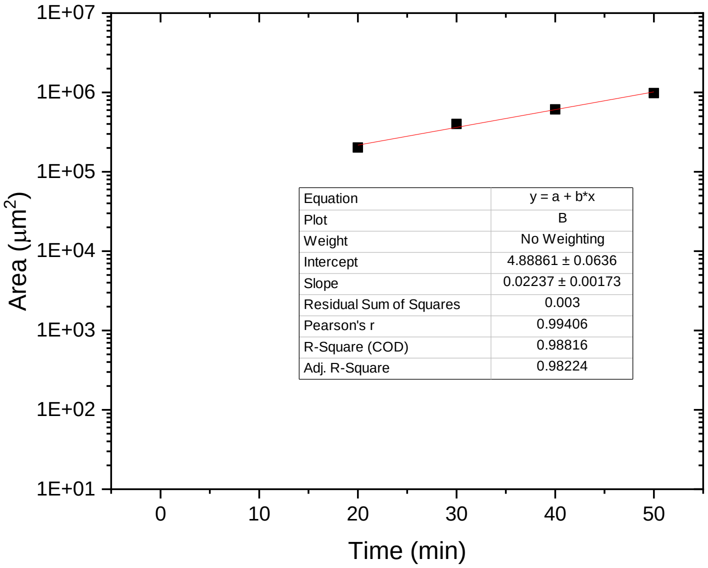

Figure S10. Linear fit of area for floc at different time using a chitosan+PAC mixture and samples from Tamazula River (70 NTU).
